# Biphenyl/PCB Degrading *bph* Genes of Ten Bacterial Strains Isolated from Biphenyl-Contaminated Soil in Kitakyushu, Japan: Comparative and Dynamic Features as Integrative Conjugative Elements (ICEs)

**DOI:** 10.3390/genes10050404

**Published:** 2019-05-27

**Authors:** Jun Hirose, Hidehiko Fujihara, Takahito Watanabe, Nobutada Kimura, Hikaru Suenaga, Taiki Futagami, Masatoshi Goto, Akiko Suyama, Kensuke Furukawa

**Affiliations:** 1 Department of Applied Chemistry, Faculty of Engineering, University of Miyazaki, Miyazaki 889-2192, Japan; 2 Department of Food and Fermentation Sciences, Faculty of Food and Nutrition Sciences, Beppu University, Beppu 874-8501, Japan; fujihara@nm.beppu-u.ac.jp (H.F.); aksuyama@nm.beppu-u.ac.jp (A.S.); kfurukaw@nm.beppu-u.ac.jp (K.F.); 3 Research Institute for Sustainable Humanosphere, Kyoto University, Uji 611-0011, Japan; takahito@rish.kyoto-u.ac.jp; 4 Bioproduction Research Institute, National Institute of Advanced Industrial Science and Technology (AIST), Tsukuba 305-8566, Japan; n-kimura@aist.go.jp; 5 Biotechnology Research Institute for Drug Discovery, National Institute of Advanced Industrial Science and Technology (AIST), Tokyo 135-0064, Japan; suenaga-hikaru@aist.go.jp; 6 Education and Research Center for Fermentation Studies, Faculty of Agriculture, Kagoshima University, Kagoshima 890-0065, Japan; futagami@chem.agri.kagoshima-u.ac.jp; 7 Faculty of Agriculture, Saga University, Saga 840-8502, Japan; mgoto@cc.saga-u.ac.jp

**Keywords:** biphenyl, *bph* gene, integrative conjugative element, genome sequence

## Abstract

We sequenced the entire genomes of ten biphenyl/PCB degrading bacterial strains (KF strains) isolated from biphenyl-contaminated soil in Kitakyushu, Japan. All the strains were Gram-negative bacteria belonging to β- and γ-proteobacteria. Out of the ten strains, nine strains carried a biphenyl catabolic *bph* gene cluster as integrative conjugative elements (ICEs), and they were classified into four groups based on the structural features of the *bph* genes. Group I (five strains) possessed *bph* genes that were very similar to the ones in *Pseudomonas* *furukawaii* KF707 (formerly *Pseudomonas pseudoalcaligenes* KF707), which is one of the best characterized biphenyl-utilizing strains. This group of strains carried salicylate catabolic *sal* genes that were approximately 6-kb downstream of the *bph* genes. Group II (two strains) possessed *bph* and *sal* genes similar to the ones in KF707, but these strains lacked the *bphX* region between *bphC* and *bphD*, which is involved in the downstream catabolism of biphenyl. These *bph-sal* clusters in groups I and II were located on an integrative conjugative element that was larger than 110 kb, and they were named ICE*_bph-sal_*. Our previous study demonstrated that the ICE*_bph-sal_* of *Pseudomonas putida* KF715 in group II existed both in an integrated form in the chromosome (referred to as ICE*_bph-sal_*KF715 (integrated)) and in a extrachromosomal circular form (referred to as ICE*_bph-sal_* (circular)) (previously called pKF715A, 483 kb) in the stationary culture. The ICE*_bph-sal_* was transferred from KF715 into *P. putida* AC30 and *P. putida* KT2440 with high frequency, and it was maintained stably as an extrachromosomal circular form. The ICE*_bph-sal_*KF715 (circular) in these transconjugants was further transferred to *P*. *putida* F39/D and then integrated into the chromosome in one or two copies. Meanwhile, group III (one strain) possessed *bph* genes, but not *sal* genes. The nucleotide sequences of the *bph* genes in this group were less conserved compared to the genes of the strains belonging to groups I and II. Currently, there is no evidence to indicate that the *bph* genes in group III are carried by a mobile element. Group IV (two strains) carried *bph* genes as ICEs (59–61 kb) that were similar to the genes found in Tn*4371* from *Cupriavidus oxalacticus* A5 and ICE_KKS102_*4677* from the *Acidovorax* sp. strain KKS102. Our study found that *bph* gene islands have integrative functions, are transferred among soil bacteria, and are diversified through modification.

## 1. Introduction

To date, a number of biphenyl-utilizing bacteria have been isolated and characterized in terms of the degradation of polychlorinated biphenyls (PCBs), which are serious environmental contaminants that are prevalent worldwide [1,2,3]. These strains include both Gram-negative and Gram-positive bacteria. Biphenyl catabolic enzymes co-metabolize certain PCBs into chlorobenzoic acids. It is well documented that PCB degradation is highly dependent on chlorine substitutions, such as the number and positions of the substituted chlorine [4]. Degradation capabilities are also strain dependent. For the first time, biphenyl catabolic *bph* genes were cloned from *Pseudomonas furukawaii* KF707 [5]. Since then, *bph* genes were cloned from various strains, including both Gram-negative and Gram-positive bacteria, and then they were analyzed in detail [3]. These studies indicated that some strains possessed *bph* genes that were very similar to the ones in KF707 in terms of gene organization and nucleotide sequences, although some strains possessed *bph* gene clusters that were different from KF707 and diversified from each other [3]. Some *bph* genes are located on chromosomes, whereas others are present on plasmids. The *bph* genes of *C. oxalacticus* A5 (formerly *Ralstonia* sp. strain A5) [6] and *Acidovorax* sp. KKS102 [7] are located on the ICEs. Gram-positive *Rhodococcus jostii* RHA1 possesses multiple *bph* genes on large linear plasmids [8,9].

**Figure 1:**
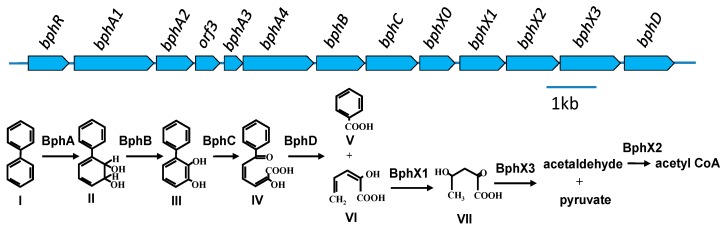
Catabolic pathway of the biphenyl degradation and organization of the *bph* gene cluster in *P. furukawaii* KF707. Compounds: I, biphenyl; II, 2,3-dihydroxy-4-phenylhexa-4,6-diene (dihydrodiol compound); III, 2,3-dihydroxybiphenyl; IV, 2-hydroxy-6-oxo-6-phenylhexa-2,4-dienoic acid (biphenyl *meta*-cleavage compound: HOPD); V, benzoic acid; VI, 2-hydroxypenta-2,4-dienoic acid; VII, 4-hydroxy-2-oxovalerate. Enzymes: BphA1-BphA2-BphA3-BphA4, biphenyl dioxygenase; BphB, dihydrodiol dehydrogenase; BphC, 2,3-dihydroxybiphenyl dioxygenase; BphX0, glutathione *S*-transferase; BphX1, 2-hydroxypenta-2,4-dienoate hydratase; BphX2, acetaldehyde dehydrogenase (acylating); BphX3, 4-hydoxy-2-oxovalerate aldolase; BphD, 2-hydroxy-6-oxo-6-phenylhexa-2,4-dieonic acid hydrolase. The BphR protein, belonging to the GntR family, is a transcriptional regulator involved in the expression of *bphR* and *bphX0X1X2X3D*. The function of *orf3* remains unclear.

The typical *bph* gene cluster shown in KF707 is composed of *bphRA1A2A3A4BCX0X1X2X3D* (Figure 1) [3]. Briefly, the biphenyl dioxygenase is a multi-component enzyme encoded by *bphA1A2A3A4,* and it catalyzes the initial oxygenation of biphenyl, converting the biphenyl into dihydrodiol, where *bphA1* and *bphA2* encode a large and a small subunit of the terminal dioxygenase, respectively. *bphA3* encodes ferredoxin, and *bphA4* encodes ferredoxin reductase. The dihydrodiol compound is then converted to a dihydroxy-compound by the dehydrogenase encoded by the *bphB*. The dihydroxy-compound is then degraded into 2-hydroxy-6-oxo-6-phenylhexa-2,4-dienoic acid by the ring-cleavage dioxygenase encoded by the *bphC*. Then, the ring *meta*-cleavage compound is degraded into benzoic acid and 2-hydroxypenta-2,4-dienoic acid by the hydrolase (encoded by the *bphD*). BphX1X2X3 is responsible for the further degradation of 2-hydroxypenta-2,4-dienoic acid into acetyl CoA. These structural *bph* genes are regulated by the *bphR* located on the *bph* gene cluster [10,11] Among these *bph* genes, *bphA1* is critically important for substrate specificity, i.e., the biodegradation capability for various aromatic compounds, including PCBs [12,13,14].

Previously, we isolated more than ten biphenyl-utilizing bacterial strains (KF strains) from biphenyl-contaminated soil in Kitakyushu, Japan [15]. Among these KF strains, we determined the complete nucleotide sequence of the *P. putida* KF715 genome [16], which revealed five replicons: one circular chromosome and four plasmids. Southern blot analysis indicated that the majority of the KF715 cell population carried the *bph-sal* cluster on its chromosome. However, a small population of cells carried the cluster on a huge extrachromosomal circular element called pKF715A (483 kb). In addition, this element carried the *oriT* sequence, the *repA* gene involved in replication, the conjugal transfer gene (*tra*), and the partitioning gene (*par*). In this study, we were interested in how the KF strains isolated from the same location carried *bph* gene clusters along with other catabolic genes. We performed whole genome sequencing of these strains, anticipating that their genome information would shed light on the diversity and evolution of biphenyl-utilizing bacteria. Our results indicated that specific DNA blocks, including the *bph* gene cluster, were integrated within glycine tRNA (tRNA-Gly) genes and that some blocks contained an integrase gene, illustrating that certain *bph* gene islands had integrative functions.

## 2. Materials and Methods

### 2.1. Bacterial Strains and Cultivation

The bacterial strains (KF strains) used in this study are presented in Table 1. These biphenyl/PCB degrading strains were isolated at the biphenyl-manufacturing factory in Kitakyushu, Japan [15], and were deposited to the National Biological Resource Center (NBRC). Strains KF701, KF703, KF707, KF708, and KF712 were renamed based on the 16S rRNA sequence [17,18,19,20,21]. *P. putida* AC30Bph+ and *P. putida* KT2440Bph+ were obtained through conjugation with *P. putida* KF715, and these two transconjugants grew on biphenyl as a sole source of carbon and energy, as described in Reference [22]. *P. putida* F39/D is a mutant of toluene utilizing *P. putida* F1 [23], in which the *todD* gene is defective. This strain was used as a recipient for the conjugation experiments. The growth of the KF strains and the transconjugants was examined on a basal salt agar medium with biphenyl and various aromatic compounds as described in Reference [5].

### 2.2. Genome Sequencing and Computational Analysis

The whole genome sequences of the KF strains were determined by the National Institute of Technology and Evaluation (NITE), using a combination of shotgun sequencing on a 454 GS FLX+ system (Roche, Basel, Switzerland) and paired-end sequencing on a HiSeq sequencing system (Illumina, San Diego, CA, USA) as previously reported in Reference [17]. The reads obtained by the two systems were assembled using the Newbler version 2.8 (Roche). The draft sequence data of the *P. furukawaii* KF707 and *P. putida* KF715 were further completed using the GenoFinisher computer program (http://www.ige.tohoku.ac.jp/joho/gf_e/). Remaining gaps between the contigs were closed using polymerase chain reaction (PCR) amplification and DNA sequencing with standard Sanger technology. The genome sequences were annotated using the RAST (Rapid Annotation using Subsystem Technology) server [30]. The identification of the coding genes was checked using a BLAST search (http://www.ncb.gov/BLAST/). Sequence comparison was performed using EasyFig Ver. 2.1 [31], and the map was generated using drawGeneArrows3 (http://www.ige.tohoku.ac.jp/joho/labhome/tool.html). The whole genome sequences of the 10 strains were deposited to the DDBJ/EMBL/GenBank under the accession numbers presented in Table 1. The nucleotide sequences of the integrative conjugative elements in KF701, KF702, KF703, KF707, KF708, KF710, KF712, and KF716 were deposited separately. Their accession numbers were LC469607, LC469608, LC469609, LC469610, LC469611, LC469612, LC469613, and LC469614, respectively.

### 2.3. Phylogenetic Analysis and Gene Alignment

The nucleotide sequences were aligned computationally using the ClustalW algorithm as in Reference [32]. Phylogenetic trees were generated using the neighbor-joining method with the Mega 6.0 program [33]. The trees were evaluated through bootstrap resampling (500 replicates).

### 2.4. Conjugation Experiments

Transfer of the Bph^+^ phenotype (*P. putida* AC30Bph+ and *P. putida* KT2440Bph+) by conjugation into the recipient cells (*P. putida* F39/D) was carried out through filter mating as described in Reference [22].

### 2.5. DNA Manipulation

DNA isolation, Southern blot analysis, PCR, DNA sequencing, and other DNA manipulations were performed according to standard procedures as described in Reference [34]. Pulsed-field gel electrophoresis was performed in accordance with the manufacturer’s instructions (Bio-Rad Laboratories, Hercules, CA, USA).

## 3. Results

### 3.1. KF Strains and their Genomic Features

**Figure 2:**
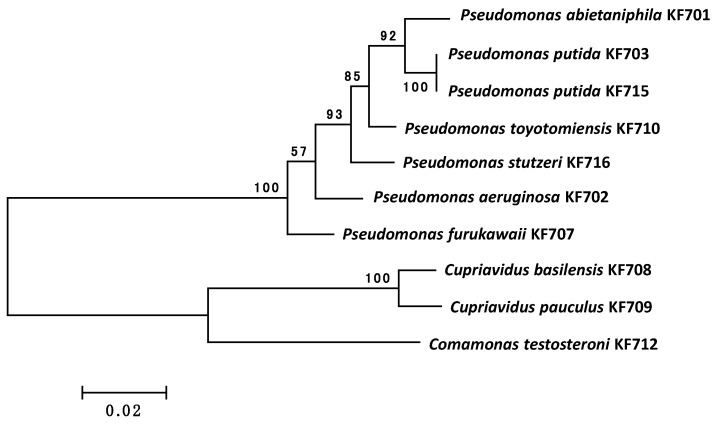
16S rRNA phylogenetic trees of the ten KF strains. The multiple alignment outputs were used to generate neighbor-joining phylogenetic trees using MEGA 6.0 [33]. The bar indicates expected nucleotide substitutions per site. Numbers indicate the percentage occurrence of the branch in the bootstrapped trees on 500 replicates.

In this study, we found that various types of *bph* genes were present in the ten biphenyl/PCB degrading strains isolated from the biphenyl-contaminated soil. Figure 2 shows the 16S rRNA phylogenetic tree of the ten strains. Among the ten strains, seven belonged to the *Pseudomonas* genus, two strains to *Cupriavidus* spp*.,* and one to *Comamonas* spp. These were all Gram-negative bacteria belonging to β-proteobacteria (*Comamonas* and *Cupriavidus*) and γ-proteobacteria (*Pseudomonas*). The complete genome sequences were determined for *P. furukawaii* KF707 and *P. putida* KF715. KF707 possessed one circular chromosome of 6,242,949 bp and one plasmid (pKF707) of 59,819 bp. On the other hand, *P. putida* KF715 possessed one circular chromosome of 6,583,376 bp and four plasmids as previously reported in Reference [16]. The total length of the contigs (total number of contigs, >500 bp) of the remaining strains were as follows: *P. abietaniphila* KF701, 6,886,250 bp (140) [17]; *P. aeruginosa* KF702, 7,167,540 bp (91) [24]; *P. putida* KF703, 6,434,897 bp (135) [18]; *C. basilensis* KF708, 7,826,077 bp (62) [20]; *C. pauculus* KF709, 6,826,799 bp (227) [24]; *P. toyotomiensis* KF710, 5,596,721 bp (29) [27]; *C. testosteroni* KF712, 5,890,323 bp (97) [21]; and *P. stutzeri* KF716, 4,188,013 bp (30) [29].

All ten strains possessed the *bph* genes, while seven strains possessed the salicylate catabolic *sal* genes. The *bph-sal* cluster was localized on the chromosome in KF707. The same cluster was located on the chromosome in the majority of the KF715 cells and also existed as an extrachromosomal circular form (483,376 bp) as well in the minor part of the cells in the stationary phase culture [16]. Based on the features of the *bph* genes, we classified the ten strains into four groups. Group I (five strains) possessed the *bph-sal* cluster that was almost identical to that of KF707, where the *sal* genes were localized approximately 6-kb downstream of the *bph* genes. This group included strains KF702, KF703, KF707, KF710, and KF716. The *bph* gene cluster of this group was composed of *bphRA1A2A3A4BCX0X1X2X3D* (11.2 kb), which encodes the catabolic enzymes that degrade biphenyl into benzoate and acetyl CoA via the *meta*-cleavage pathway. Group II (strains KF701 and KF715) possessed the *bph-sal* cluster that was similar to that of KF707, although the *bphX* region (3.5 kb) that is involved in the metabolism of 2-hydroxypenta-2,6-dienoic acid to acetyl CoA (Figure 1, lower pathway of biphenyl metabolism) was deleted as described in Reference [22]. The structural features of the *bphRABCD* (7.8 kb) and the flanking regions, including the *sal* genes (10.2 kb), were almost identical between KF701 and KF715, indicating that the *bph-sal* clusters of these two strains were horizontally transferred to one another. Group III (KF709) possessed the *bph* genes, but not the adjacent *sal* genes as in the case of *Burkholderia xenovorans* LB400 [35], another well-characterized PCB-degrader. Overall alignment of the *bph* gene cluster in KF709 was similar to that of group I, but each *bph* component of the *bph* genes was relatively low. Group IV (KF708 and KF712) possessed *bph* genes similar to the genes found in Tn*4371* from *C. oxalacticus* A5 [6], and the ones in ICE_KKS102_*4677* from the *Acidovorax* sp. strain KKS102 [7]. Some rearrangements of the *bph* gene cluster were observed compared to that in groups I–III as described below. This group of strains did not carry the *sal* genes.

All the strains possessed the catabolic genes of benzoate, the lower pathway intermediate of biphenyl catabolism. Strains KF701, KF702, KF703, KF707, KF710, and KF715 possessed multiple benzoate catabolic genes that encoded both the extradiol cleavage *meta*-pathways (*bza*) and intradiol cleavage *ortho-*pathways (*ben*). KF708, KF709, and KF712 possessed the *box* genes encoding the benzoate catabolic pathway via benzoyl-CoA. Besides the *bph*, *sal*, and benzoate catabolic (*bza*, *ben*, and *box*) genes, these ten strains possessed catabolic genes for various aromatic compounds. Eight strains (KF701, KF703, KF707, KF708, KF710, KF712, KF715, and KF716) possessed a phenol-catabolic *dmp* gene cluster [36]. Five strains (KF702, KF703, KF707, KF710 and KF715) had protocatechuate catabolic *pca* genes. Five strains (KF703, KF707, KF708, KF709 and KF715) possessed phenylacetate catabolic *paa* genes. Other than the aromatic catabolic genes, many strains carried heavy metal-resistant genes. Six strains (KF701, KF702, KF708, KF710, KF712, and KF715) possessed a cluster of *mer* genes that were responsible for the resistance of inorganic mercury [37]. All of the ten strains possessed putative *czc* genes involved in the resistance of cobalt, zinc, and cadmium [38]. In KF708 and KF709, more than ten putative *czc* gene clusters were present. Arsenate-resistant genes were present in all the ten strains. These results, except for the arsenate-resistant genes, are summarized in Table S1.

### 3.2. Comparison of the bph Genes

**Figure 3:**
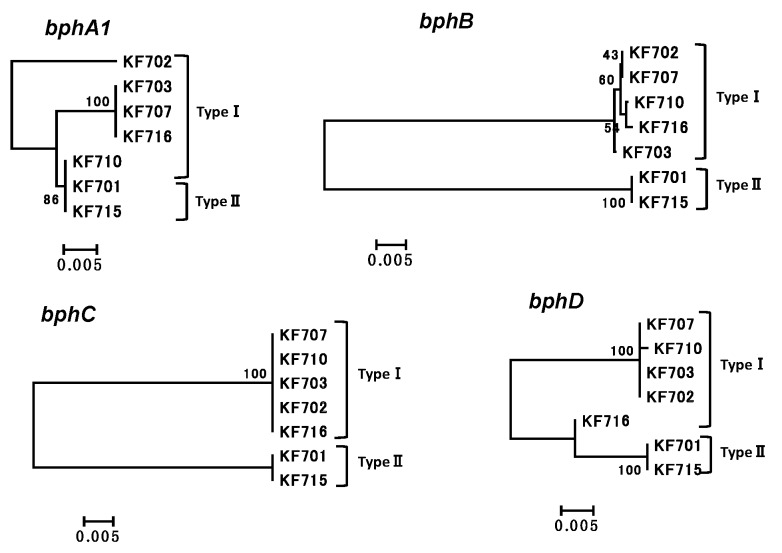
Phylogenetic tree of *bphA1*, *bphB*, *bphC*, and *bphD* of KF strains in groups I and II (types I and II *bph* genes). The multiple alignment outputs were used to generate neighbor-joining phylogenetic trees using MEGA 6.0 [33]. The bar indicates expected nucleotide substitutions per site.

**Figure 4:**
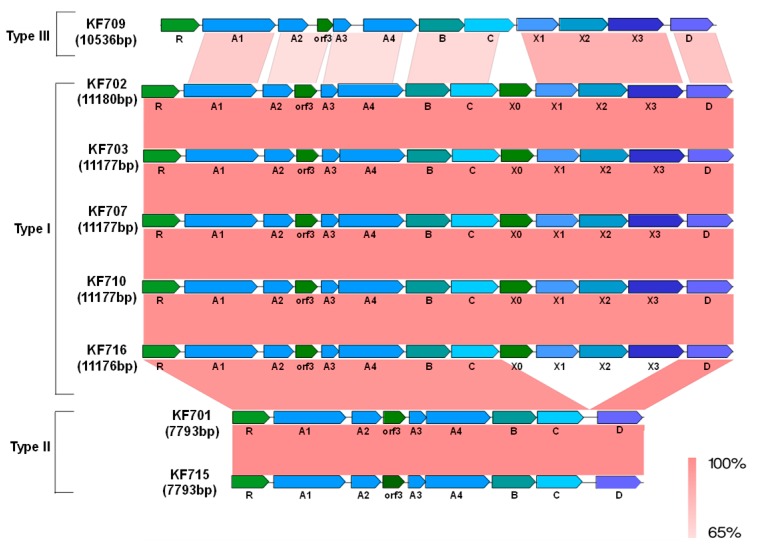
Comparison of the *bph* gene clusters of KF strains belonging to groups I–III (types I–III). The shading in pink to red shows the identity (65–100%) of the gene clusters as indicated at the bottom of the figure. R, *bphR*; A1, *bphA1*; A2, *bphA2*; A3, *bphA3*; A4, *bphA4*; B, *bphB*; C, *bphC*; D, *bphD*; X0, *bphX0*, X1, *bphX1*; X2, *bphX2*; and X3, *bphX3*.

The identities (percent) of the nucleotide sequences of the *bph* genes in groups I–IV strains (named as types I–IV *bph* genes, respectively) are shown in Tables S2–S13. The phylogenetic trees of *bphA1*, *bphB*, *bphC*, and *bphD* belonging to types I and II are shown in Figure 3. A comparative analysis of the nucleotide sequences of each gene revealed that all the *bph* genes were almost identical in types I and II (96.7–100%), except for that of the *bphX* region (3.5 kb) between *bphC* and *bphD*, which was missing in the *bph* gene cluster of type II (Figure 4).

The *bphA1*, *bphA2*, *bphA3*, *bphA4*, *bphB*, and *bphC* genes of KF716 were almost identical to that of the type I *bph* gene. However, the 3’-terminus of the *bphX3* gene (754 bp) and 5’-terminus of the *bphD* gene (260 bp) of KF716 were different from that of type I (Figure S1a,b), and the 5’-terminus of the *bphD* gene (260 bp) of KF716 was almost identical to that of the type II *bph* gene. These genetic features are reflected in the phylogenetic tree of the *bphD* (Figure 3). Thus, the *bph* genes of KF716 showed the structural features of both groups I and II.

The KF709 (group III strain) possessed a type III *bph* gene similar to the one in group I, but the respective *bph* genes were less conserved and rearranged (Figure 4). The nucleotide sequences of the KF709 *bph* genes had less than 72.7% identities compared to types I and II in the *bphABC* region (5.9 kb). However, the *bphX1, X2, X3* genes and *bphD* gene were more identical (88.6–90.6% in *bphX1* and 74.3–84.9% in *bphX2, bphX2, bphX3,* or *bphD*) (Tables S2–S13). The *bphX0* encoding putative glutathione *S*-transferase was not present between the *bphC* and *bphX1,* but it was located downstream of the tRNA-Gly gene (data not shown). *orf3* was present between the *bphA2* and *bphA3* of types I–III. Despite the conserved feature of *orf3*, its function remains unclear [3].

**Figure 5:**
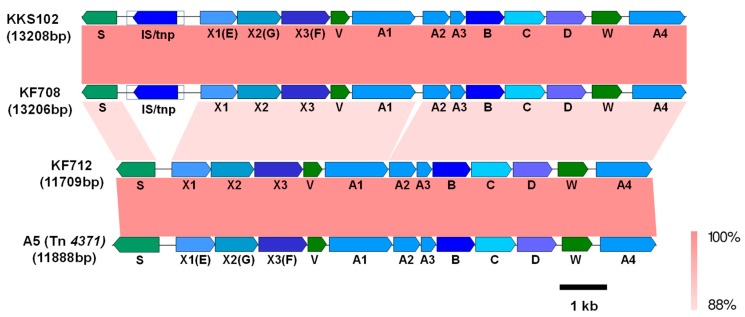
Comparison of the *bph* genes of *Cupriavidus basilensis* KF708 and *Comamonas testosteroni* KF712 belonging to group IV (type IV) to those of *Acidovorax* sp. KKS102 and *Cupriavidus oxalacticus* A5. The identities (88–100%) between the gene clusters are shown by shading in pink to red as indicated at the right bottom of the figure. E, *bphE*; F, *bphF*; G, *bphG*; S, *bphS*; V, *bphV*; W, *bphW*; IS, insertion sequence; tnp, transposase. The *bphE, bphF, and bphG of* KKS102/Tn*4371* are homologous genes of *bphX1, bphX3,* and *bphX2* of KF708/KF712, respectively. The other signs are defined in the legend in Figure 4.

The genetic features of KF708 and KF712 in the group IV strain *bph* genes (Type IV *bph* gene) were very different to types I and II, yet they were similar to those of the *Acidovorax* sp. strain KKS102 and *C. oxalacticus* A5 (Figure 5). The *bph* gene cluster of this type was composed of *bphSX1X2X3*(*V*)*A1A2A3BCD*(*W*)*A4*. Thus, the *bphX1X2X3* region was located upstream of the *bphA1A2A3BCD* region, and *bphA4* was present just downstream of the *bphD* (Figure 5). The insertion sequence (1190 bp) and the transposase gene (IS/tnp) were present between the *bphS* and *bphX1* of KF708 and KKS102 [39], but these were not present in KF712 and *C. oxalacticus* A5. The identity of the KF708 and KF712 *bph* genes of type IV was compared to types I and II (Tables S2–S13). The nucleotide sequences of *bphA1, bphA2, bphA3, bphA4, bphB, bphC*, and *bphD* were conserved between types I and IV; however, the identities were less than 77%. Two unidentified gene components, *bphV* and *bphW*, were present in all the type IV *bph* genes, but they were not present in types I–III. The type IV *bph* genes possessed a transcriptional regulator, *bphS* [39]. It was reported that *bphS* acts as a repressor, whereas the *bphR* of Type I acts as an activator for biphenyl catabolism. The functions of *bphS* and *bphR* oppose each other, although they belong to the same GntR family [10,39].

### 3.3. ICE_bph-sal_ in KF Strains Belonging to Groups I and II

**Figure 6:**
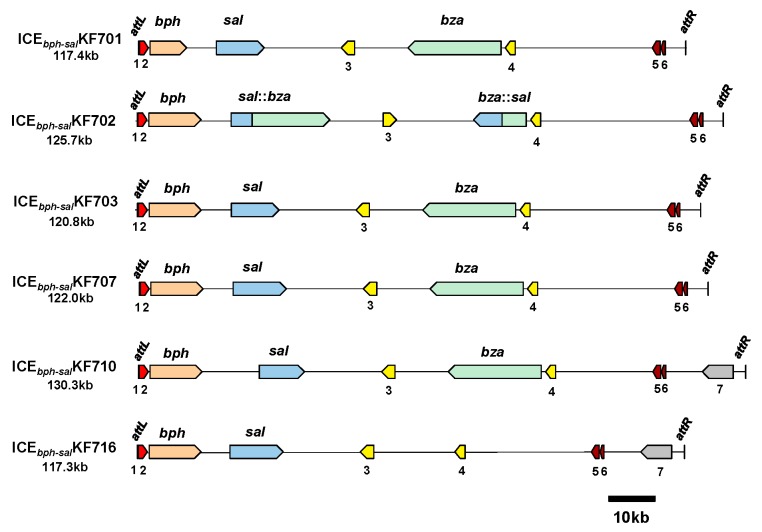
Organization of the ICE*_bph-sal_* in KF strains of group I and KF701 of group II. ICE*_bph-sal_*KF701, ICE*_bph-sal_*KF702, ICE*_bph-sal_*KF703, ICE*_bph-sal_*KF707, and ICE*_bph-sal_*KF710 carry the *int* gene, *bph* genes, *sal* genes, and *bza* genes. ICE*_bph-sal_*KF716 carries the *int* gene, *bph*, and *sal* genes, but not the *bza* genes. The *sal* genes and *bza* genes in ICE*_bph-sal_*KF702 are recombined. 1, tRNA-Gly(CCC) genes (partial); 2, *int* genes; 3, VirB4 components of the type IV secretory pathway; 4, VirD4 component of the type IV secretory pathway; 5, *parB* genes; 6, *parA* genes; and 7, putative multidrug efflux pumps. The other, undefined gene components are not shown in the figure.

**Figure 7:**
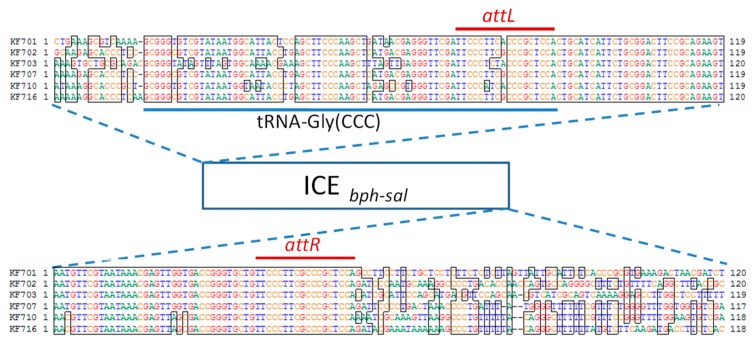
Integration sites of ICE*_bph-sal_* in the KF strains of group I and KF701 of group II. The *attL* (18 bp) site, including 18 bp of the 3’ end of tRNA-Gly gene, and *attR* (18 bp) sites are indicated.

The genome sequence analysis of KF701, KF702, KF703, KF707, KF715, and KF716 revealed highly conserved large “genomic islands” that included the *bph-sal* genes adjacent to the tRNA-Gly(CCC) gene (Figure 6). There was an 18 bp direct repeat (5’-TTCCC(T/A)(T/C)(C/T)(G/A)CCCGCTCCA-3’) on the border of the conserved region and the non-conserved region (*attL* and *attR*, Figure 7). The *attL* included 18 bp of the 3ʹ end of the tRNA-Gly(CCC) gene. These 18 bp direct repeat sequences could be generated by the integration of genomic islands into the chromosome. In strain KF707, *attL* was followed by a phage-related integrase (*int*) gene. The *bph* cluster was located just downstream of the *int* gene, followed by the *sal* gene cluster approximately 6-kb downstream, and by the *bza* gene approximately 49-kb downstream. The *attR* site was located downstream of the *bza* gene. Thus, the genomic island was estimated to be 122.0 kb in size (Figure 6). Genes identified as the VirB4 component (ATPase) and VirD4 component (coupling protein) of the type IV secretory pathway [40] were located 40-kb and 80-kb downstream of the tRNA-Gly(CCC) gene, respectively. The *parA* and *parB* genes encoding the replication partition proteins were present near the right end, and were proposed to act as a stabilization system for the maintenance of mobile elements in the bacterial genomes [41]. These structures corresponded to the common backbone of many integrative conjugative elements (ICEs) [42], such as ICE*clc* from *Pseudomonas knackmussii* B13 [43], which carries the catabolic genes of chlorocatechols, the tyrosine integrase gene, and type IV secretory machinery. We designated it as ICE*_bph-sal_*KF707. Within this element, many putative mobile protein genes were present surrounding the *sal* gene cluster. To note, five IS genes at the upstream region and two IS genes at the downstream region were identified. ICE*_bph-sal_* was observed in other KF strains of group I and KF701 of group II. An ICE including the *bph* and *sal* gene clusters of *P. aeruginosa* KF702, named ICE*_bph-sal_*KF702, was calculated to be 126.7 kb (Figure 6). Likewise, the ICE*_bph-sal_*KF703 of *P. putida* KF703, ICE*_bph-sal_*KF710 of *P. toyotomiensis* KF710, and ICE*_bph-sal_*KF716 of *P. stutzeri* KF716 were calculated to be 120.8 kb, 130.3 kb, and 117.3 kb, respectively. The ICE*_bph-sal_*KF701 of *P. abietaniphila* KF701 in the group II strain was 117.4 kb in size. As in the case of ICE*_bph-sal_*KF707, ICE*_bph-sal_*KF701, ICE*_bph-sal_*KF702, ICE*_bph-sal_*KF703, and ICE*_bph-sal_*KF710 contained three gene clusters of *bph*, *sal*, and *bza*. ICE*_bph-sal_*KF716 contained *bph* and *sal* genes, but not the *bza* genes. Sequence comparison revealed an inversion in ICE*_bph-sal_*KF702. The *sal:bza* and *bza:sal* fusion gene clusters were found, in which half parts of the *sal* genes and the *bza* genes were replaced with each other. These fusion genes were likely generated via homologous recombination between the *sal* and the *bza* genes. ICE*_bph-sal_*KF710 and ICE*_bph-sal_*KF716 contained the 5-kb region encoding putative multidrug efflux pumps, but ICE*_bph-sal_*s from the remaining KF strains was deficient of the corresponding region. Thus, ICE*_bph-sal_* in groups I and II contained highly conserved nucleotide sequences larger than 110 kb, which were larger than many other ICEs found in bacterial strains to date [44].

We previously reported that KF715 harbors an approximately 90-kb conjugative *bph-sal* gene cluster in the chromosome [22], and that the cluster could be transferred to *P. putida* AC30 and *P. putida* KT2440 with very high frequency. Whole genome sequencing of the KF715 studied here revealed that the *bph-sal* cluster was located on a huge 483-kb extrachromosomal element, previously designated as pKF715A [16]. An 18 bp DNA sequence identical to the 3’- end portion of a tRNA-Gly(CCC) gene as part of its attachment site (*attP*) was present just upstream of the *int* gene in this element. It was also confirmed that the bacterial integration site (*attB*) was present within the 3ʹ-end portion of a tRNA-Gly gene in the KF715 chromosome. Furthermore, the two *Spe*I digested bands, approximately 300 kb and 200 kb, hybridized with a *bphA1* probe were observed as reported [16]. Since there was only one *Spe*I site in the element, the large, faint band was matched to 314 kb of the *Spe*I digested extrachromosomal element. The second major band of 220 kb was matched to that of the *Spe*I digested chromosome of strain KF715. This suggested that the majority of the KF715 cells carried an integrated ICE*_bph-sal_*KF715, whereas fewer cells carried it as an extrachromosomal circular form. Thus, it was likely that the circular form could be obtained by recombining the *attL* site (present at the 3ʹ-end of the tRNA-Gly gene) and *attR* site (present far downstream, 483 kb from the *attL* site), forming the *attP* site.

### 3.4. ICEbph in the Group IV Strains

**Figure 8:**
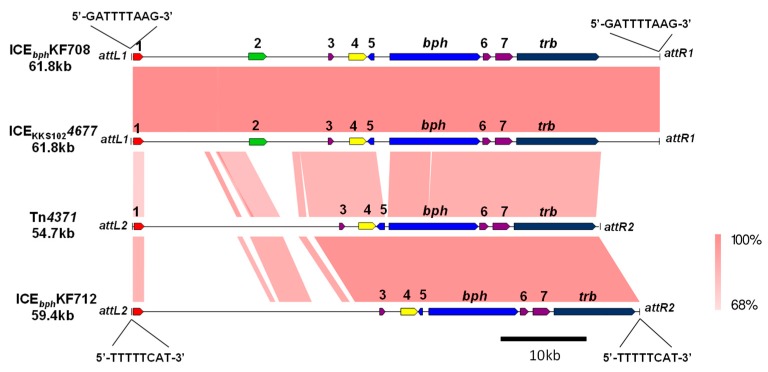
Organization of ICE*_bph_*KF708 and ICE*_bph_*KF712 in comparison to ICE_KKS102_*4677* and Tn*4371*. *attL1* and *attR1* represent integration sites for the ICE*_bph_*KF708 and ICE_KKS102_*4677. attL2* and *attR2* represent integration sites for the Tn*4371* or ICE*_bph_*KF712. 1, integrase (*int*) gene; 2, putative arsenate-resistant gene; 3, *parA* gene; 4, VirD2 component (relaxase); 5, transcriptional regulator *bphS* gene; 6, *traR* gene; and 7, *traG* gene. The shading in pink to red shows the identities (68–100%) of the gene clusters as indicated at the right bottom of the figure.

The *bph* genes of *C. basilensis* KF708 and *C. testosteroni* KF712 were also located on different types of ICEs. ICE*_bph_*KF708 (61.8 kb), including *bph* genes of KF708, was almost identical to ICE_KKS102_*4677* of the *Acidovorax* sp. strain KKS102 [7] (Figure 8). ICE*_bph_*KF708 had covalently bound ends of the conserved 5ʹ-GATTTTAAG-3’ sequence (*attL1* and *attR1*). This *att* sequence was identical to that of ICE_KKS102_*4677*. Nine nucleotide substitutions, three nucleotide deletions, and one nucleotide insertion were found in ICE*_bph_*KF708 when compared to ICE_KKS102_*4677.* The ICE*_bph_*KF712 (59.4 kb) carrying the *bph* genes of KF712 was almost identical to the Tn*4371* from *C. oxalaticus* A5, being the first ICE found carrying the *bph* gene cluster [6]. ICE*_bph_*KF712 had the covalently bound ends of the conserved *attL2* and *attR2* sequence (5’-TTTTCAT-3’). This *att* sequence was identical to that of Tn*4371*, but it was different from the *attL1* and *attR1* sequence (5’-GATTTTAAG-3’) of ICE_KKS102_*4677* and KF708. The core part, including the *bph* and *trb* gene of ICE*_bph_*KF712 in length of 33 kb, was almost identical to that of Tn*4371*, whereas the remaining part flanked by the *attL2* site was less conserved. Major parts of these ICEs shared common structures, including the *bph* gene cluster; genes encoding the replication and partition proteins; *parA*; VirD2 component (relaxase); as well as two conjugative transfer elements, the *trb* gene cluster, and the *tra* genes (Figure 8). ICE*_bph_*KF708 and ICE_KKS102_*4677* possessed a putative arsenate-resistant gene cluster at 15.5–18.5 kb downstream of the *attL* site, which encoded the transcriptional regulator, arsenate reductase, and arsenite efflux transporter. The gene cluster corresponding to them was not found in ICE*_bph_*KF712 and Tn*4371*.

### 3.5. Conjugal Transfer of Extrachromosomal ICEbph-salKF715 Into P. putida F39/D

**Figure 9:**
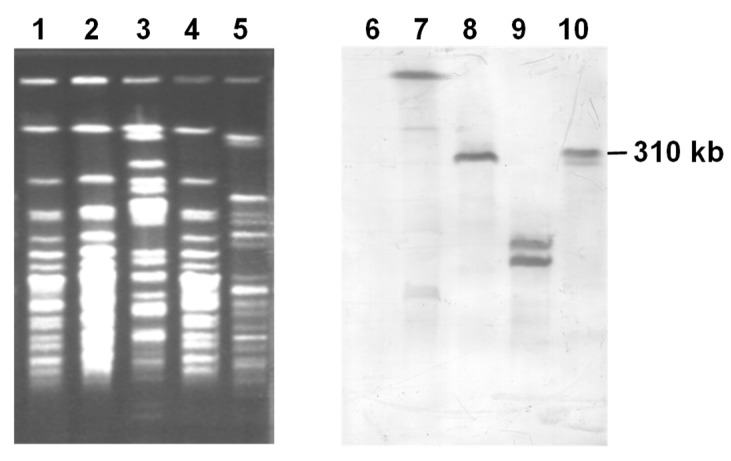
Conjugative transfer of the *bph* genes from *P. putida* AC30Bph+ and *P. putida* KT2440Bph+ into *P. putida* F39/D. Genomic DNA was digested with *Spe*I, applied to pulse field gel electrophoresis, and subjected to Southern blot analysis. The *bphA1* DNA of KF715 was used as a probe. Lanes 1 and 6, F39/D; lanes 2 and 7, F39/DBph+ transconjugant from AC30Bph+; Lanes 3 and 8, AC30Bph+; Lanes 4 and 9, F39/DBph+ transconjugant from KT2440Bph+; and Lanes 5 and 10, KT2440Bph+.

Since *P. putida* AC30 Bph+ and *P. putida* KT2440 Bph+ harbor an extrachromosomal ICE*bph-sal*KF715, from strain KF715, we tried to transfer this element into *P. putida* F39/D through filter mating. The results are shown in Figure 9. Interestingly, the Bph+ transconjugant of F39/D exhibited two bands hybridized to KF715 *bphA1* DNA when KT2440Bph+ was used as a donor strain of pKF715A. On the other hand, only a single band was detected when AC30Bph+ was used as a donor strain. These hybridized bands were all different in size to that of the 310-kb *Spe*I fragment of pKF715A, indicating that the extrachromosomal ICE*_bph-sal_*KF715 of the two donor strains were transferred into the recipient strain F39/D and then integrated into the chromosomes at different loci.

## 4. Discussion

Bacteria evolve through a variety of genetic events, such as mutations, intergenomic shuffling, and horizontal gene transfer. ICEs are being identified in increasing numbers via bacterial genomic analysis. Genomic islands, including ICEs, are discrete DNA segments and play an important role in bacterial evolution [42,45,46]

In this study, we revealed that *bph* genes differentially existed in ten biphenyl/PCB degrading strains isolated from biphenyl-contaminated soil. The types I and II *bph* genes of KF strains belonging to groups I and II were very similar in terms of gene organization and nucleotide sequences, except that the *bphX* region was missing in the type II *bph* gene cluster. The ICE structures carrying the *bph* genes were identified in several strains. Typical ICE*_bph-sal_* of approximately 120 kb was observed in the group I strains and KF701 of group II, and they were accompanied by the *sal* genes, approximately 6-kb downstream of the *bph* genes. The KF715 strain of group II carried *bph* and *sal* gene clusters in the chromosome and also on a plasmid (pKF715A, 483 kb) [16]. ICEs are thought to reintegrate into the recipient’s chromosome immediately after transfer. However, a recent study suggested that certain ICEs, such as ICE*Bs1* from *Bacillus subtilis* [47] and SXT/R391 ICEs from *Vibrio cholerae* [48], are capable of autonomous replication. Transconjugants of *P. putida* AC30Bph+ and *P. putida* KT2440Bph+ carry the *bph-sal* cluster as an extrachromosomal circular form [16]. The mobile element carrying the *bph-sal* cluster replicates autonomously like plasmid, maintained stably, and consists of genes sharing homologies to components of the DNA replication and stabilization machinery.

The first example of ICE-harboring genes for the degradation of xenobiotic compounds is the *clc* element (ICE*clc*, 105 kb) from *P. knackmussii* B13. ICE*clc* encodes for the catabolic pathway involved in 3- and 4-chlorocatechol degradation [43,49,50,51]. Besides this catabolic gene cluster, ICE*clc* has the core genes, such as type IV secretion system-encoding genes, relaxase, and integrase. ICE*clc* can excise through recombination between short direct repeats at either end (*attL* and *attR*). The excised ICE*clc* can transfer to a new recipient cell through the conjugation apparatus and it integrates into the recipient’s chromosome between the 18-bp sequence at the 3’ end of the tRNA-Gly gene on the chromosome (*attB*) and the identical sequence on the excised ICE*clc* (*attP*), thereby restoring the tRNA-Gly gene. Both excision and integration are mediated by the IntB13 integrase. The ICE*_bph-sal_*s of group I (strains KF702, KF703, KF707, KF710, and KF716) were related to ICE*clc* in terms of the conjugation apparatus and the core gene set. However, the genetic organizations of ICE*_bph-sal_*s are different from those of ICE*clc*. The integrases in the group I ICE*_bph-sal_* were almost identical (~99% in amino acid sequences), but it was as low as 59% between that of ICE*_bph-sal_* and ICE*clc*. Such low identity of the integrases between ICE*_bph-sal_* and ICE*clc* reflected the differences of the insertion sites. The ICE*_bph-sal_*s of group I were inserted at the 3′- end of the tRNA-Gly gene (76 bp in length), which carries the CCC anticodon. ICE*clc* inserts into a number of tRNA-Gly genes, but only the genes which carry the GCC anticodon [51]. It should also be noted that the ICE*_bph-sal_* in strains belonging to groups I and II are present in *Pseudomonas* spp., whereas other strains than *Pseudomonas* possess different ICE*_bph_* as seen in group IV strains, indicating that ICE*_bph-sal_* have restricted host ranges.

In this study, we found that various types of *bph* genes are present in ten different strains isolated from the same soil sample. Several lines of evidence suggested that many *bph* genes in these strains were present on the chromosome as an integrated form. However, it is also true that certain ICE*_bph-sal_* is present stably as a plasmid. ICE*_bph-sal_*KF715 was stably maintained as a circular form in the two transconjugants, *P. putida* AC30Bph+ and *P. putida* KT2440Bph+. On the other hand, ICE*_bph-sal_*KF715 (circular) from *P. putida* AC30Bph+ and *P. putida* KT2440Bph+ seemed to integrate differently in another recipient *P. putida* F39/D. When *P. putida* AC30Bph+ was used as a donor strain, the largest *Spe*I DNA fragment of the F39/DBph+ transconjugant was hybridized with the KF715 *bphA1* probe (Figure 9). When *P. putida* KT2440Bph+ was used as a donor strain, two copies of the *bphA1* DNA were detected in the F39/DBph+ transconjugant strain at different positions (Figure 9). Investigations are currently underway to reveal how the ICE*_bph-sal_*KF715 is integrated in the genome of the F39/DBph+ transconjugants. The ICE*clc* of the *P. knackmussii* strain B13 was transferred by conjugation and integrated into two nonadjacent sites on the chromosome of toluene utilizing *P. putida* F1 [49]. Our repeated attempts to conjugally transfer the ICE*_bph-sal_* of other strains than KF715 have not been successful. This may be due to a lack of expression of the integrase genes or mutations in certain gene(s) involved in the excision or conjugal transfer.

ICE*_bph_*KF708 and ICE*_bph_*KF712 were found in *Cupriavidus* and *Comamonas*, respectively. ICE*_bph_*KF708 was almost identical to the ICE_KKS102_*4677* from *Acidovorax* sp. KKS102, and they had several nucleotide differences, indicating that this type of ICE*_bph_* could be transferred between *Cupriavidus* and *Acidovorax*. The right wing corresponding to the core legion (33 kb) of ICE*_bph_*KF712 and Tn*4371* is highly conserved, but the left wing is diversified (Figure 8). This indicated that these two ICE*_bph_* were rearranged in the left wing. ICE*_bph-sal_* (from the groups I and II strain) and ICE*_bph_* (from the group IV strain) are typical ICEs, which possess type IV secretion machinery. However, there are few relationships between ICE*_bph-sal_* and ICE*_bph_* in terms of gene organization, nucleotide sequence, and size (Figure 6, Figure 8). No significant identity was detected between the integrases of ICE*_bph_* and ICE*_bph-sal_*. The region encoding two conjugative transfer components, the *tra*/*trb* genes adjacent to the *bph* gene cluster, was found in ICE*_bph_*. The corresponding gene clusters were not found in ICE*_bph-sal_*. Gene components downstream of the *sal* gene cluster are likely to be involved in conjugative transfer in ICE*_bph-sal_*; however, they have not been identified because of the lack of reliable homologous genes that are identified as conjugative transfer components in public databases. Although ICE*_bph-sal_* and ICE*_bph_* possess common genetic components involved in biphenyl catabolism, their platforms are different.

The biphenyl degrading bacteria are considered to be responsible for lignin degradation at the final stage. Lignin is a complex compound based on the phenylpropane structure and contains a variety of biphenyl related molecules. Thus, the *bph* genes could be very ancient and distributed across a wide range of soil bacteria. Mobilization of the *bph* genes in soil bacteria can be achieved through various mobile genetic elements, including ICEs, transposons, and plasmids. It is highly conceivable that these genes can be modified and rearranged in different ways in new host cells. The results in this study provide a better understanding as to how soil bacteria exchange genetic islands involved in the catabolism of aromatic compounds, as well as how such genes are rearranged and modified in the natural environment.

## Figures and Tables

**Table 1:** Biphenyl/PCB degrading KF strains used in this study.

Strain	NBRC Number	DDBJ/EMBL/GenBank Accession Number	References
*Pseudomonas abietaniphila* KF701	110664	B BQJ01000001-BBQJ01000140	[15,17]
*Pseudomonas aeruginosa* KF702	110665	B BQK01000001-BBQK01000091	[15,24]
*Pseudomonas putida* KF703	110666	BBQL01000001-BBQL01000135	[15,18]
*Pseudomonas furukawaii* KF707	110670	AP014862	[15,19,25]
*Cupriavidus basilensis* KF708	110671	B BQM01000001-BBQM01000062	[15,20]
*Cupriavidus pauculus* KF709	110672	BBQN01000001-BBQN01000227	[15,26]
*Pseudomonas toyotomiensis* KF710	110674	BBQO01000001-BBQO01000029	[15,27]
*Comamonas testosteroni* KF712	110673	BBQP01000001-BBQP01000097	[15,21]
*Pseudomonas putida* KF715	110667	AP015029, AP015030-AP015033	[15,16,22,28]
*Pseudomonas stutzeri* KF716	110668	BBQQ01000001-BBQQ01000030	[29]

## Supplementary Materials

The following tables and figure are available online at https://www.mdpi.com/2073-4425/10/5/404/s1. Table S1: Distribution of the catabolic genes for aromatic compounds and the heavy metal resistance genes in the biphenyl/PCB degrading KF strains. Tables S2–S13: Identity (%) of nucleotide sequence of *bphR, bphA1, bphA2, bphA3, bphA4, bphB, bphC, bphX0, bphX1, bphX2, bphX3,* and *bphD.* Figure S1: Comparison of the *bphX3* (a) and *bphD* (b) genes belonging to types I and II.
