# Supplementary material for: Biphenyl/PCB Degrading bph Genes of Ten Bacterial Strains Isolated from Biphenyl-Contaminated Soil in Kitakyushu, Japan: Comparative and Dynamic Features as Integrative Conjugative Elements (ICEs)
Source: Genes (Basel). 2019 May 27;10(5):404. doi: 10.3390/genes10050404 (PMC6563109; doi:10.3390/genes10050404)
Supplement: Supplementary file 1 [file genes-10-00404-s001.pdf]

Table S1. Distribution of the catabolic genes for aromatic compounds and the heavy metal resistance genes in the biphenyl/PCB degrading KF strains.

**KF strains**	***bph***	***sal***	***bza***	***ben***	***box***	***dmp***	***pca***	***paa***	***mer***	***czc***
701	+	+	+	+	-	+	-	-	+	+
702	+	+	+	+	-	-	+	-	+	+
703	+	+	+	+	-	+	+	+	-	+
707	+	+	+	+	-	+	+	+	-	+
708	+	-	-	-	+	+	-	+	+	+
709	+	-	-	+	+	-	-	+	-	+
710	+	+	+	+	-	+	+	-	+	+
712	+	-	-	-	+	+	-	-	+	+
715	+	+	+	+	-	+	+	+	+	+
716	+	+	-	+	-	+	-	-	-	+

Table S2. Identity (%) of the nucleotide sequence of bphR.

	**703**	**707**	**710**	**716**	**701**	**715**	**709**	**708**	**712**
702	99.9	99.9	99.9	99.9	99.9	99.9	61.1	<60.0	<60.0
703	-	100.0	100.0	100.0	100.0	100.0	61.1	<60.0	<60.0
707	-	-	100.0	100.0	100.0	100.0	61.1	<60.0	<60.0
710	-	-	-	100.0	100.0	100.0	61.1	<60.0	<60.0
716	-	-	-	-	100.0	100.0	61.1	<60.0	<60.0
701	-	-	-	-	-	100.0	61.1	<60.0	<60.0
715	-	-	-	-	-	-	61.1	<60.0	<60.0
709	-	-	-	-	-	-	-	<60.0	<60.0
708	-	-	-	-	-	-	-	-	88.5

Table S3. Identity (%) of the nucleotide sequence of bphA1.

	703	707	710	716	701	715	709	708	712
702	96.7	96.7	97.5	96.7	97.5	97.5	71.9	76.2	76.1
703	-	100.0	99.0	100.0	99.0	99.0	72.7	76.5	77.0
707	-	-	99.0	100.0	99.0	99.0	72.7	76.5	77.0
710	-	-	-	99.0	100.0	100.0	72.3	76.5	77.0
716	-	-	-	-	99.0	99.0	72.7	76.5	77.0
701	-	-	-	-	-	100.0	72.3	76.5	76.8
715	-	-	-	-	-	-	72.3	76.5	76.8
709	-	-	-	-	-	-	-	74.5	74.1
708	-	-	-	-	-	-	-	-	92.2

Table S4. Identity (%) of the nucleotide sequence of bphA2.

	703	707	710	716	701	715	709	708	712
702	99.7	99.7	99.5	99.5	99.5	99.5	66.7	69.2	66.7
703	-	100.0	99.8	99.8	99.8	99.8	66.7	68.7	67.0
707	-	-	99.8	99.8	99.8	99.8	66.7	69.0	67.0
710	-	-	-	100.0	100.0	100.0	66.0	68.2	67.4
716	-	-	-	-	100.0	100.0	66.0	69.2	67.4
701	-	-	-	-	-	100.0	66.0	69.2	67.4
715	-	-	-	-	-	-	66.0	69.2	67.4
709	-	-	-	-	-	-	-	66.8	68.3
708	-	-	-	-	-	-	-	-	87.3

Table S5. Identity (%) of the nucleotide sequence of bphA3.

	703	707	710	716	701	715	709	708	712
702	100.0	100.0	100.0	100.0	100.0	100.0	67.0	71.0	70.4
703	-	100.0	100.0	100.0	100.0	100.0	67.0	71.0	70.4
707	-	-	100.0	100.0	100.0	100.0	67.0	71.0	70.4
710	-	-	-	100.0	100.0	100.0	67.0	71.0	70.4
716	-	-	-	-	100.0	100.0	67.0	71.0	70.4
701	-	-	-	-	-	100.0	67.0	71.0	70.4
715	-	-	-	-	-	-	67.0	71.0	70.4
709	-	-	-	-	-	-	-	65.4	63.9
708	-	-	-	-	-	-	-	-	93.9

Table S6. Identity (%) of the nucleotide sequence of bphA4.

	703	707	710	716	701	715	709	708	712
702	100.0	100.0	100.0	100.0	92.7	92.7	62.2	<60.0	<60.0
703	-	100.0	100.0	100.0	92.7	92.7	62.2	<60.0	<60.0
707	-	-	100.0	100.0	92.7	92.7	62.2	<60.0	<60.0
710	-	-	-	100.0	92.7	92.7	62.2	<60.0	<60.0
716	-	-	-	-	92.7	92.7	62.2	<60.0	<60.0
701	-	-	-	-	-	100.0	62.6	<60.0	<60.0
715	-	-	-	-	-	-	62.6	<60.0	<60.0
709	-	-	-	-	-	-	-	<60.0	<60.0
708	-	-	-	-	-	-	-	-	87.6

Table S7. Identity (%) of the nucleotide sequence of bphB.

	**703**	**707**	**710**	**716**	**701**	**715**	**709**	**708**	**712**
702	99.9	100.0	99.9	99.8	92.0	92.0	71.3	73.8	74.9
703	-	99.9	99.8	99.6	92.1	92.1	71.4	73.9	75.0
707	-	-	99.9	99.8	92.0	92.0	71.3	73.8	74.9
710	-	-	-	99.9	91.8	91.8	71.2	73.7	74.9
716	-	-	-	-	92.0	92.0	71.3	73.8	75.1
701	-	-	-	-	-	100.0	70.2	72.9	74.9
715	-	-	-	-	-	-	70.2	72.9	74.9
709	-	-	-	-	-	-	-	68.9	70.1
708	-	-	-	-	-	-	-	-	92.1

Table S8. Identity (%) of the nucleotide sequence of *bphC*.

	**703**	**707**	**710**	**716**	**701**	**715**	**709**	**708**	**712**
702	100.0	100.0	100.0	100.0	92.6	92.6	<60.0	68.7	69.6
703	-	100.0	100.0	100.0	92.6	92.6	<60.0	68.7	69.6
707	-	-	100.0	100.0	92.6	92.6	<60.0	68.7	69.6
710	-	-	-	100.0	92.6	92.6	<60.0	68.7	69.6
716	-	-	-	-	92.6	92.6	<60.0	68.7	69.6
701	-	-	-	-	-	100.0	<60.0	67.9	69.4
715	-	-	-	-	-	-	<60.0	67.9	69.4
709	-	-	-	-	-	-	-	<60.0	60.2
708	-	-	-	-	-	-	-	-	92.9

Table S9. Identity (%) of the nucleotide sequence of bphX0.

	**703**	**707**	**710**	**716**
702	100.0	100.0	100.0	100.0
703	-	100.0	100.0	100.0
707	-	-	100.0	100.0
710	-	-	-	100.0

Table S10. Identity (%) of the nucleotide sequence of bphX1 (bphE).

	**703**	**707**	**710**	**716**	**709**	**708**	**712**
702	100.0	100.0	100.0	95.4	90.6	<60.0	<60.0
703	-	100.0	100.0	95.4	90.6	<60.0	<60.0
707	-	-	100.0	95.4	90.6	<60.0	<60.0
710	-	-	-	95.4	90.6	<60.0	<60.0
716	-	-	-	-	88.6	<60.0	<60.0
709	-	-	-	-	-	<60.0	<60.0
708	-	-	-	-	-	-	91.5

Table S11. Identity (%) of the nucleotide sequence of bphX2 (bphG).

10	703	707	710	716	709	708	712
702	100.0	100.0	100.0	96.6	84.5	64.2	63.4
703	-	100.0	100.0	96.6	84.5	64.2	63.4
707	-	-	100.0	96.6	84.5	64.2	63.4
710	-	-	-	96.6	84.5	64.2	63.4
716	-	-	-	-	84.9	63.9	63.3
709	-	-	-	-	-	64.9	63.5
708	-	-	-	-	-	-	83.3

Table S12. Identity (%) of the nucleotide sequence of bphX3 (bphF).

	703	707	710	716	709	708	712
702	100.0	100.0	100.0	91.2	80.6	66.1.	65.5
703	-	100.0	100.0	91.2	80.6	66.1	65.5
707	-	-	100.0	91.2	80.6	66.1	65.5
710	-	-	-	91.2	80.6	66.1	65.5
716	-	-	-	-	77.8	64.8	65.5
709	-	-	-	-	-	66.7	66.7
708	-	-	-	-	-	-	85.1

Table S13. Identity (%) of the nucleotide sequence of bphD.

	703	707	710	716	701	715	709	708	712
702	100.0	100.0	99.9	97.2	96.2	96.2	75.2	75.2	75.6
703	-	100.0	99.9	97.2	96.2	96.2	75.2	75.2	75.6
707	-	-	99.9	97.2	96.2	96.2	75.2	75.2	75.6
710	-	-	-	97.1	96.1	96.1	75.0	75.2	75.5
716	-	-	-	-	99.0	99.0	74.8	75.3	76.2
701	-	-	-	-	-	100.0	74.3	75.0	75.7
715	-	-	-	-	-	-	74.3	75.0	75.7
709	-	-	-	-	-	-	-	74.4	74.1
708	-	-	-	-	-	-	-	-	91.8

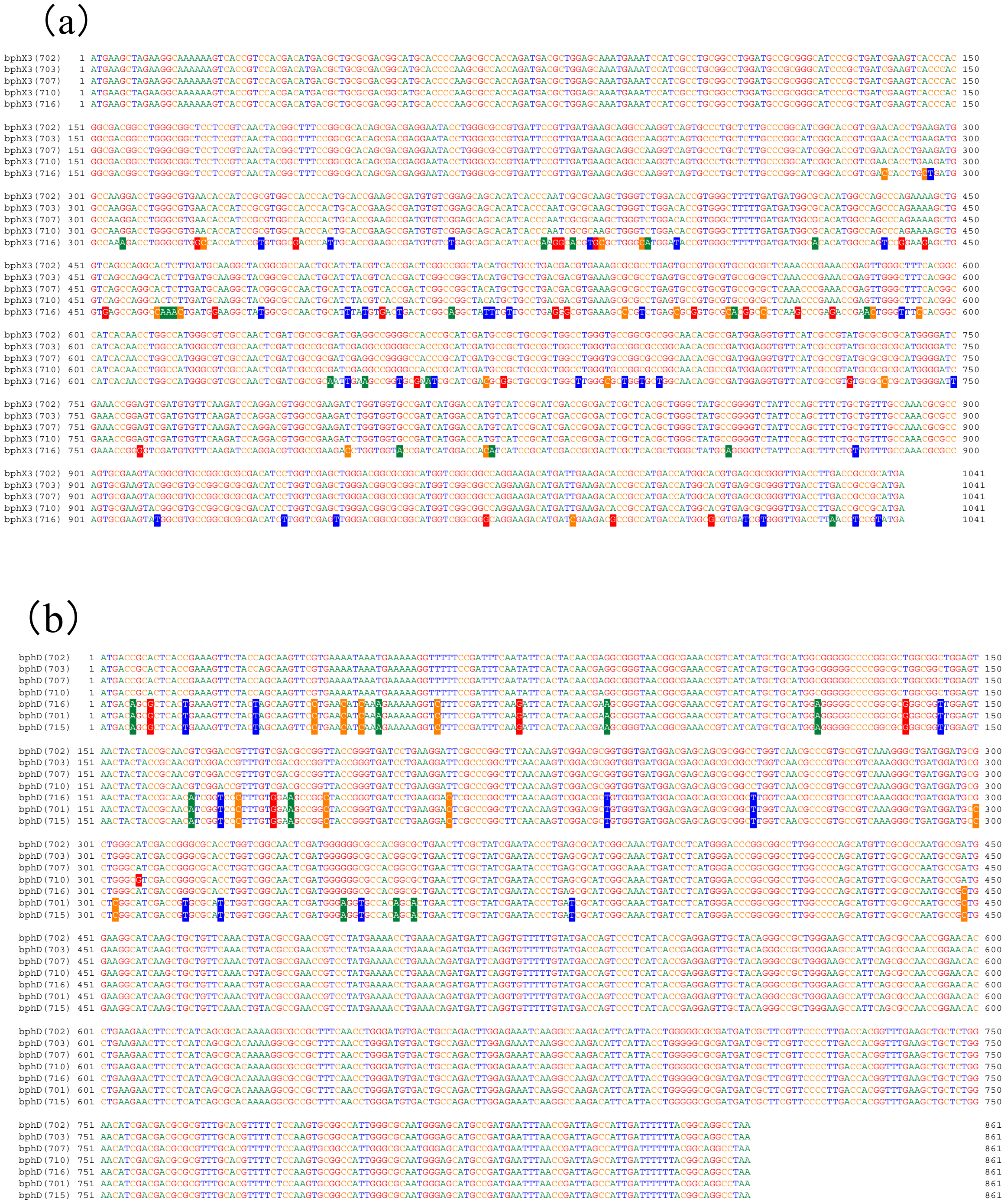

Figure S1. Comparison of the *bphX3* (a) and *bphD* (b) genes belonging to type I and type II. Note that the *bph* genes of KF716 show structural features of both type I and type II as described in the text.
